# Molecular and genomic investigation of an urban outbreak of dengue virus serotype 2 in Angola, 2017–2019

**DOI:** 10.1371/journal.pntd.0010255

**Published:** 2022-05-18

**Authors:** Zoraima Neto, Pedro A. Martinez, Sarah C. Hill, Domingos Jandondo, Julien Thézé, Marinela Mirandela, Renato Santana Aguiar, Joilson Xavier, Cruz dos Santos Sebastião, Ana Luísa Micolo Cândido, Filipa Vaz, Gisel Reyes Castro, Joana Paula Paixão, Nicholas J. Loman, Philippe Lemey, Oliver G. Pybus, Jocelyne Vasconcelos, Nuno Rodrigues Faria, Joana de Morais

**Affiliations:** 1 Instituto Nacional de Investigação em Saúde (INIS), Ministry of Health, Luanda, Angola; 2 Department of Pathobiology and Population Sciences, Royal Veterinary College, London, United Kingdom; 3 Department of Zoology, University of Oxford, Oxford, United Kingdom; 4 Université Clermont Auvergne, INRAE, Clermont-Ferrand, France; 5 Departamento de Genética, Ecologia e Evolução, Instituto de Ciências Biológicas, Universidade Federal de Minas Gerais, Belo Horizonte, Brazil; 6 Laboratório de Genética Celular e Molecular, ICB, Universidade Federal de Minas Gerais, Belo Horizonte, Brazil; 7 World Health Organization Angola, Luanda, Angola; 8 Institute of Microbiology and Infection, University of Birmingham, Birmingham, United Kingdom; 9 Department of Microbiology, Immunology and Transplantation, Rega Institute, KU Leuven, Leuven, Belgium; 10 Instituto de Medicina Tropical, Faculdade de Medicina da Universidade de São Paulo, São Paulo, Brazil; 11 MRC Centre for Global Infectious Disease Analysis, J-IDEA, Imperial College London, London, United Kingdom; University of Texas Medical Branch, UNITED STATES

## Abstract

**Background:**

The transmission patterns and genetic diversity of dengue virus (DENV) circulating in Africa remain poorly understood. Circulation of the DENV serotype 1 (DENV1) in Angola was detected in 2013, while DENV serotype 2 (DENV2) was detected in 2018. Here, we report results from molecular and genomic investigations conducted at the Ministry of Health national reference laboratory (INIS) in Angola on suspected dengue cases detected between January 2017 and February 2019.

**Methods:**

A total of 401 serum samples from dengue suspected cases were collected in 13 of the 18 provinces in Angola. Of those, 351 samples had complete data for demographic and epidemiological analysis, including age, gender, province, type of residence, clinical symptoms, as well as dates of onset of symptoms and sample collection. RNA was extracted from residual samples and tested for DENV-RNA using two distinct real time RT-PCR protocols. On-site whole genome nanopore sequencing was performed on RT-PCR+ samples. Bayesian coalescent models were used to estimate date and origin of outbreak emergence, as well as population growth rates.

**Results:**

Molecular screening showed that 66 out of 351 (19%) suspected cases were DENV-RNA positive across 5 provinces in Angola. DENV RT-PCR+ cases were detected more frequently in urban sites compared to rural sites. Of the DENV RT-PCR+ cases most were collected within 6 days of symptom onset. 93% of infections were confirmed by serotype-specific RT-PCR as DENV2 and 1 case (1.4%) was confirmed as DENV1. Six CHIKV RT-PCR+ cases were also detected during the study period, including 1 co-infection of CHIKV with DENV1. Most cases (87%) were detected in Luanda during the rainy season between April and October. Symptoms associated with severe dengue were observed in 11 patients, including 2 with a fatal outcome. On-site nanopore genome sequencing followed by genetic analysis revealed an introduction of DENV2 Cosmopolitan genotype (also known as DENV2-II genotype) possibly from India in or around October 2015, at least 1 year before its detection in the country. Coalescent models suggest relatively moderately rapid epidemic growth rates and doubling times, and a moderate expansion of DENV2 in Angola during the studied period.

**Conclusion:**

This study describes genomic, epidemiological and demographic characteristic of predominately urban transmission of DENV2 in Angola. We also find co-circulation of DENV2 with DENV1 and CHIKV and report several RT-PCR confirmed severe dengue cases in the country. Increasing dengue awareness in healthcare professional, expanding the monitorization of arboviral epidemics across the country, identifying most common mosquito breeding sites in urban settings, implementing innovative vector control interventions and dengue vaccination campaigns could help to reduce vector presence and DENV transmission in Angola.

## Author summary

Angola has experienced dengue, yellow fever and Zika virus outbreaks between 2013–2017. To improve the detection of arbovirus outbreaks in Angola, we set up the National Arbovirus Laboratory of Surveillance Programme at the National Institute of Health Research (INIS), Ministry of Health, Angola, which officially kick-started arbovirus surveillance activities in early 2017. In this study we tested samples collected from patients with suspected dengue cases between January 2017 to February 2019. We report molecular and genomic findings on DENV2 transmission in Angola. On-site whole genome sequencing and subsequent phylogenetic analysis indicate a single introduction of the DENV2 Cosmopolitan genotype (also known as DENV2-II genotype) from Southern Asia, probably as a result of international travel, followed by moderate virus population growth. In Angola, most detections occurred in urban areas, although we detected DENV2 in 5 of the 18 provinces in Angola. This work emphasizes the importance of maintaining an active arbovirus surveillance programme throughout the country to investigate DENV transmission patterns and evaluate the risk of hyperendemicity in Angola.

## Introduction

About half of the world’s population are at risk of dengue disease, of which a large proportion lives in Africa [1,2]. Dengue disease is caused by the dengue virus (DENV) which has an enveloped 10.7kb single-stranded RNA virus genome. DENV is a member of the *Flavivirus* genus, which also includes yellow fever virus (YFV) and Zika virus (ZIKV). DENV is mainly transmitted by the anthropophilic *Aedes aegypti* and *Aedes albopictus* mosquito vectors [3]. After an extensive global expansion in recent decades, these mosquitoes can now be found in urban and peri-urban areas with tropical and sub-tropical climates around the world [4]. While most infections with DENV are asymptomatic, symptomatic disease is typically characterized by fever, arthralgia, severe headache, myalgia, vomiting and rash. Occasionally, infection causes a potentially lethal complication called severe dengue that is characterized by increased vascular permeability, which can lead to fluid accumulation, haemorrhage, and shock [3].

DENV can be classified in four genetically distinct virus clades that are named as serotypes (DENV1 to DENV4). Infection with one serotype confers lifelong immunity to that serotype and temporary (0.5 to 2 years) immunity to other serotypes [5,6]. However, infection with a heterotypic serotype increases the risk of developing severe dengue disease [7]. Increases in global travel have facilitated the increased transmission and co-circulation (hyperendemicity) of DENV serotypes [8,9]. The first epidemic of severe dengue was described in the Philippines in 1953. Since then, severe dengue has been observed throughout Asia, Pacific Islands and the Americas [10].

In Africa, reports of severe dengue are rare [11–13]. Although the 4 DENV serotypes are known to circulate in Africa [14], little is known about the true burden of dengue transmission in the continent. This is in part due to the limited availability of robust serological and molecular diagnostics for routine disease surveillance in several African settings. The lack of data from the African continent complicates the generation of quality epidemiological and genetic information that could inform on the patterns and drivers of dengue transmission [15]. Improved surveillance and laboratory diagnosis of dengue cases in Africa is thus a key priority to assess the global epidemiology and transmission dynamics of dengue virus serotypes [16].

The Republic of Angola, which has over 32 million inhabitants, is located in southwestern Africa bordering the Democratic Republic of Congo in the north, Zambia in the east and Namibia in the south. DENV circulation in Angola was first evidenced indirectly in the 1980s by infected travellers returning from Angola to the Netherlands [17]. In 2013 Angola reported its first locally acquired DENV cases [18–21]. During the 2013 epidemic, around 10% of case and random cluster participants in Luanda, Angola’s capital city, had evidence of recent DENV infection [18]. That year, returning travellers from 5 countries over 4 continents acquired DENV infections in Angola [21] caused by DENV1 genotype V [20]. Subsequent phylogenetic analysis of the 2013 outbreak in Luanda revealed that this virus lineage had most likely been caused by an endemic DENV1 virus lineage circulating in West Africa [19]. Subsequently, an investigation of 47 patients returning from Angola to Portugal also identified two DENV2 cases, one co-infection with DENV1 and DENV3, and one case of DENV4 [22]. Also, in 2013 DENV4 was identified in a second traveller returning from Angola to Portugal [23].

Recent studies indicate that the *Aedes aegypti* vector is widely present in all municipalities of Luanda province, the capital of Angola [24,25]. Moreover, the recent circulation of YFV in 2016 with confirmed cases in 13 of the 18 provinces in the country [26], the local transmission of ZIKV between 2016 and 2018 [27] and the indirect evidence of chikungunya virus [28] further corroborate the existence of suitable environmental conditions conductive for the epidemic spread of arboviruses in Angola transmitted by *Aedes aegypti*, including DENV.

To improve the surveillance and management of dengue in Africa, it is important to generate quantitative information on DENV epidemiology and evolution. In Angola, active molecular surveillance for dengue, YFV, chikungunya and Zika viruses, began in January 2017 at the National Institute of Research in Health (INIS) in Luanda. INIS currently seeks to confirm dengue suspected cases from hospitals and clinics across Angola using real-time reverse transcription PCR (RT-PCR), and in some cases through virus whole genome sequencing. Analysis of DENV genome sequences can help to disentangle the origins of viral lineages and their temporal dynamics. Despite several logistic challenges regarding the generation of viral genome sequence data during outbreaks, particularly in low- and middle- income countries [29], in January 2018 we conducted the first on-site genomic sequencing of a single dengue virus strain in Angola [30].

Here we analysed clinical, demographic and epidemiological data from 351 suspected dengue cases sampled across 13 provinces in Angola, including cases of severe dengue in Angola. Next, we report genomic analysis of the first 25 DENV genomes generated at INIS in Angola. Using coalescent phylogenetic analysis, we estimate key epidemiological parameters such as date of introduction and doubling times of the main DENV lineage circulating during the study period. Finally, we contextualize our findings in light of recent arboviral outbreaks in Angola and discuss the intricacies of the current surveillance system.

## Results

Clinical and epidemiological data were collected and analyzed by the National Arbovirus Surveillance programme implemented in January 2017 and coordinated by INIS, Ministry of Health, Angola. Between January 2017 and February 2019, we tested samples collected from dengue suspected cases from 13 provinces across the Republic of Angola (Luanda, Benguela, Bengo, Cuanza Sul, Cuanza Norte, Huambo, Huila, Cunene, Namibe, Cabinda, Uíge, Zaire and Moxico) using molecular detection via RT-PCR. A total of 351 sera samples with complete epidemiological information from suspected dengue cases were tested for the presence of DENV, ZIKV and CHIKV RNA using RT-PCR assays [31, 32].

### Laboratory detection of DENV2, DENV1 and Chikungunya virus in Angola

**Fig 1:**
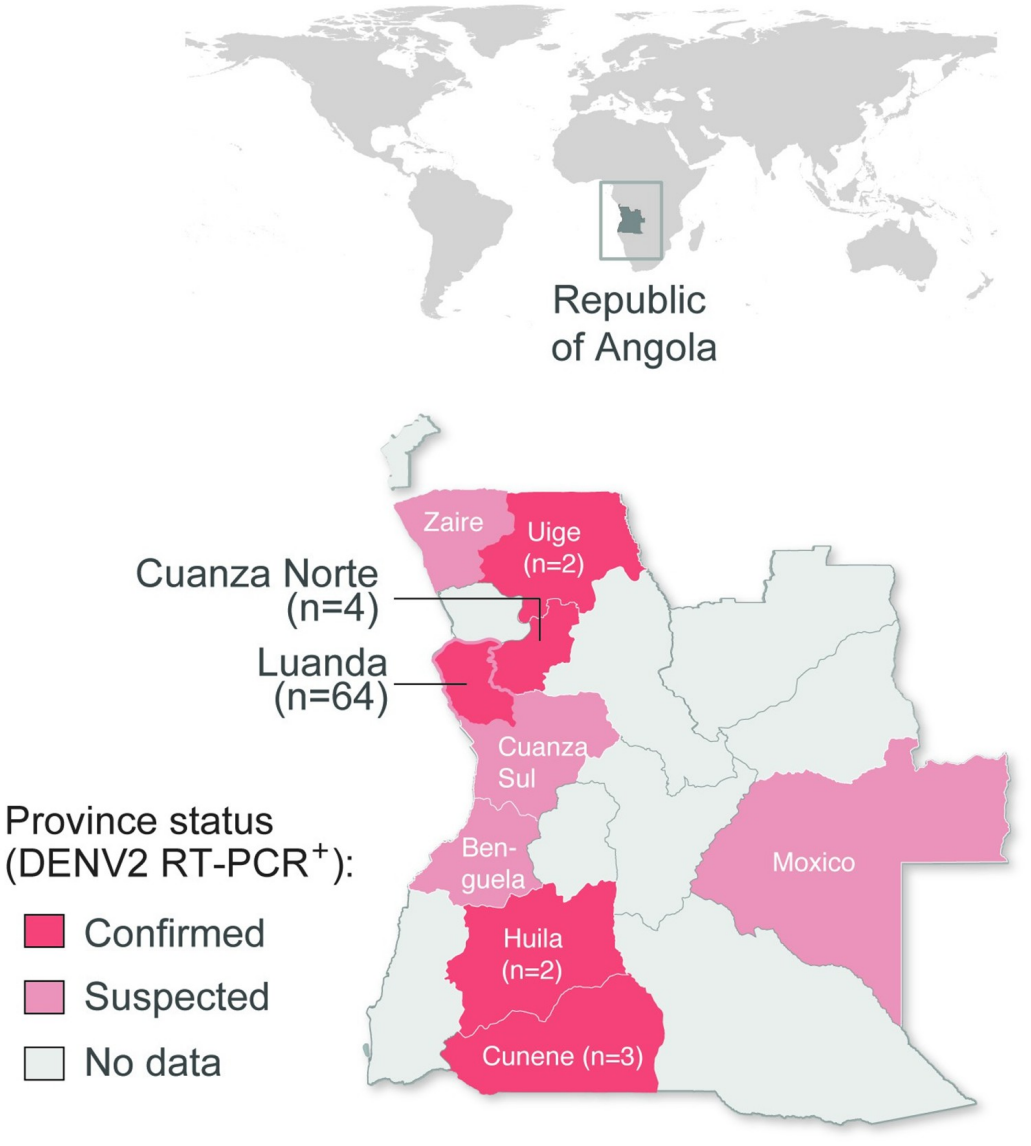
Spatial distribution of DENV2 RT-PCR confirmed cases in Angola. Upper world map shows the geopolitical location of Angola. In the lower map, the locations of DENV2 RT-PCR confirmed (dark red), suspected cases (light red). No samples were received from provinces in grey. Names of provinces with RT-PCR confirmed and suspected cases are shown.

We found a total 71 cases RT-PCR positive either for DENV (91.5%, n = 65/71), CHIKV (7%, n = 5/71) or both DENV and CHIKV (1.4%, n = 1/71) (S1 Table). We then typed DENV positive cases (n = 66) using a separate CDC DENV1-4 real time RT-PCR assay [31]. We found that most (97%, n = 64/66) DENV infections were caused by DENV2 (RT-PCR cycle threshold, mean CT = 27, range: 17 to 37). Most DENV2 infections were detected in Luanda (82.8%, n = 53/64), but we also identified DENV2 cases in Cuanza Norte (6%, n = 4/64), Cunene (4.7%, n = 3/64), Huila (3%, n = 2/64), and Uíge (3%, n = 2/64) provinces (Fig 1 and S1 Table).

A case of co-infection with DENV1 (CT = 26) and CHIKV (CT = 25) was detected in a 32-year-old male from Luanda in May 2017 presenting with fever, severe hemorrhage and fatal outcome (S1 Table). One case of DENV1 (CT = 27) was identified in a 19-year-old male patient from the Luanda province presenting with fever, myalgia, headache and arthralgia in May 2018. We also detected five CHIKV RT-PCR positive cases (mean CT = 33, range: 27 to 35) in Luanda (n = 4) and Huila (n = 1) provinces between January and April in the rainy season. No DENV3 or DENV4 infections were identified during the study period. No ZIKV infections were detected in our dataset of suspected DENV cases; Zika infections reported during the study period and subsequent outbreak investigations were reported elsewhere [27].

### Epidemiological patterns of DENV2 infections in Angola

**Fig 2:**
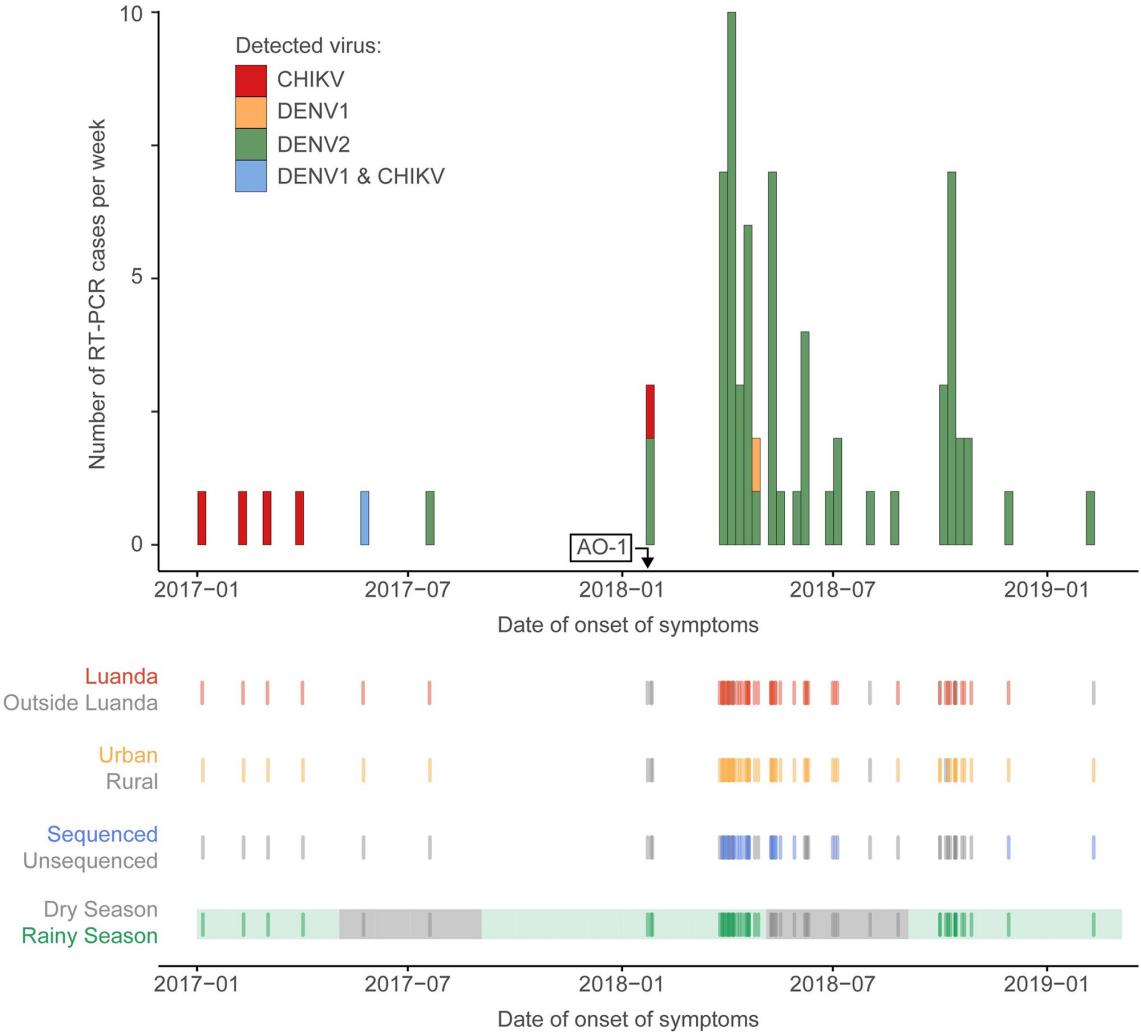
Timeseries of DENV2 RT-PCR confirmed cases Angola. Arrow indicates the date of onset of symptoms of the earliest DENV2 known case in Angola (isolate AO-1) before this study, which has previously been described in Hill et al. [30]. Rug plots show place of sample collection (Luanda/outside Luanda), residence (urban/rural), sequencing status and season corresponding to date of onset of symptoms for each DENV2 RT-PCR case positive described in S1 Table.

Most of the patients with confirmed DENV infection in our study resided in urban areas (92.4%, n = 61/66). Most DENV RT-PCR positive cases were in <18 years old individuals (51.5% n = 34/66) (S1 Table). Most PCR confirmed cases with available information regarding gender were male (60%, n = 36/65, respectively). The large majority of DENV cases (69.7%, n = 46/66) were detected in the rainy season, with the number of cases peaking between April and May 2018 (Fig 2). We have also detected cases in the dry season between June-October, possibly suggesting a potential for year-round transmission in Angola. DENV PCR confirmed cases were diagnosed more frequently in urban areas as compared to rural areas (*p*< 0.0001; odds ratio, OR = 7.9, 95% credible interval, CI = 2.77–22.8) (S2 Table).

### Clinical characteristics of DENV infections in Angola

Based on complete notification epidemiological forms for DENV RT-PCR cases, the main clinical symptoms of DENV PCR confirmed cases were fever (97%, n = 64/66), myalgias (62%, n = 41/66), and headache (57.6%, n = 38/66) and arthralgia (42%, n = 28/66) (S1 Table). Patients also reported retro-orbital pain (14%, n = 9/66), and rash (4.5%, n = 3/66) (S1 Table). Importantly, hemorrhagic signs associated with plasma leakage were found in 9 patients (14%, n = 9/66), including 8 patients from Luanda and 1 patient from the Uíge province. Of the patients presenting with severe dengue identified according to WHO criteria [10], two-thirds (n = 6/9) had one anatomic bleeding site (digestive, genitourinary, skin, or respiratory) and one-third (n = 3/9) reported bleeding from two anatomic sites. No information on platelet counts and hematocrits were available for these patients. The mean CT value for DENV2 cases with hemorrhagic signs was 26.2 (range: 20.1 to 35.8), similar to non-severe dengue infections (S1 Table). Values equal or below CT<=35 were associated with the presence of fever (*p* = 0.0459) but not with other symptoms (myalgia, retro-orbital pain, rash, and hemorrhagic signs) nor with the elapsed time between sample collection and symptom onset (not shown).

### On-site genome sequencing and genotyping of DENV2

**Fig 3:**
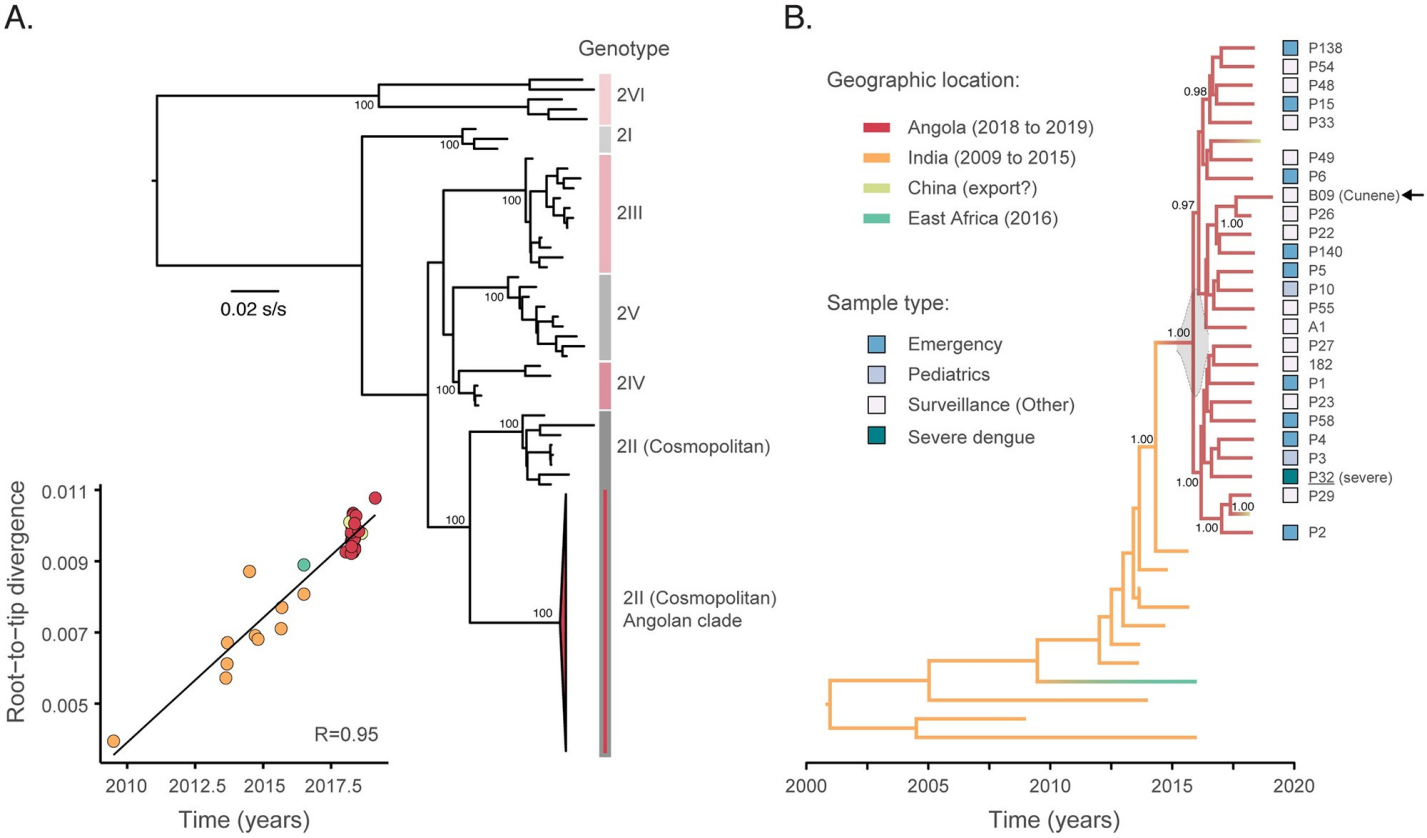
Phylogenetic analysis of the DENV2 Cosmopolitan genotype in Angola. **A**. Maximum likelihood (ML) phylogenetic tree with reference DENV2 genotype strains from [33] suggesting clustering of the Angola strains within the DENV2 Cosmopolitan genotype. Numbers indicate bootstrap support for phylogenetic clustering. The inset on the lower left corner shows the strong correlation between root-to-tip divergence and sampling dates for a ML phylogenetic tree estimated from an alignment comprising the Angola strains and the closest publicly available DENV2 Cosmopolitan genotype strains (n = 38). **B.** Dated phylogeographic tree of DENV2 Cosmopolitan genotype genomes with branches colored according to locations where strains were sampled. At the righthand side of each sequence, squares show sample type according to origin of sample. Arrow on the right highlights the most recent sample, B09 isolate, collected in the Cunene province in February 2019. s/s = substitutions per site; R = correlation coefficient.

We next attempted to generate on-site complete virus genome sequences using a previously validated nanopore sequencing approach [30]. Sequencing was conducted at INIS and attempted on a total of 25 randomly selected samples across 3 sequencing libraries. On average, we generated a median of 2,172,258 reads per library (range: 1,825,088 to 4,358,412) and obtained a median of 86.1% (range: 14–94%) of genome coverage (sites at or above 20-fold depth). We recovered a total of 25 partial to near-complete new DENV2 genomes from Angola. Using phylogenetic subtyping approaches [33], all generated genomes were classified as DENV2 genotype II (DENV2-II), also known as the DENV2 Cosmopolitan genotype (Fig 3A).

### Transmission history of DENV2 Cosmopolitan genotype in Angola

**Fig 4:**
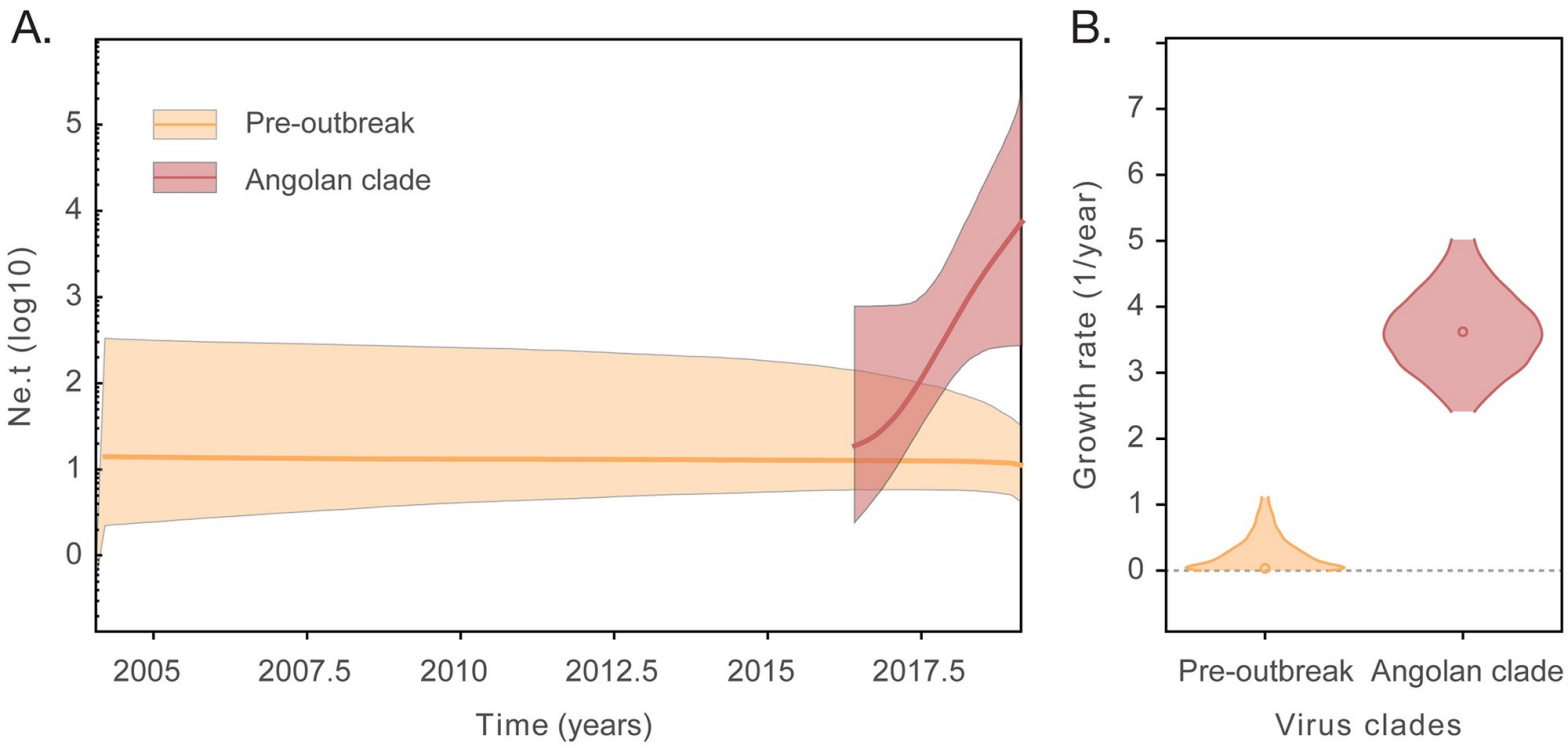
Demographic dynamics of DENV2 in Angola. **A**. Temporal changes in effective population size (Ne) multiplied by generation time (t) in logarithm scale. **B**. Logistic growth rates (yr^-1^) estimated for the pre-outbreak virus lineages (largely corresponding to samples from India, orange), and the Angolan virus lineages (red), using a nested coalescent model [34].

Our maximum likelihood phylogenetic analysis of DENV2 Cosmopolitan genotype genomes (n = 38) indicated that all strains from Angola fall within a single strongly supported monophyletic clade (bootstrap support for Angolan clade = 100%) that is closely related to sequences from India and East Africa (Fig 3). Using molecular clock analyses and a best fitting nested constant-logistic demographic model (S3 Table), we dated the common ancestor of the DENV2 Cosmopolitan genotype AO-clade in or around late October 2015 (95% BCI, April 2015 to May 2016) (Fig 3). This suggests that the DENV2 Cosmopolitan genotype may have circulated silently for over 12 months before the first confirmed DENV case in Luanda province in July 2017 (see also Fig 2). Evolutionary rates were estimated at 5.6 × 10^−4^ (95% BCI, 4.5 to 6.9 × 10^−4^) substitutions per site per year. Moreover, our best-fit constant-logistic model suggests a rate of population growth of the Angolan DENV2 outbreak at 2.59 (95% BCI, 1.57 to 3.62) new infections/individual/year (Fig 4). Given that doubling times can be obtained directly from growth rates (r) using log_2_(r), we find that the doubling time of the DENV2 Angola clade is 1.37 (95% BCI, 0.65 to 1.86) years. This reflects a moderately rapid rate of exponential population growth and/or moderate rate of geographic spread within Angola.

### Phylogeography of DENV2 Cosmopolitan genotype

To estimate the geographic origins of the DENV2 Cosmopolitan genotype epidemic in Angola, each taxon was assigned to country of sampling (Angola, China, Ethiopia, and India) and dated phylogeographic trees were estimated using a Bayesian approach that considered asymmetric location-exchange migration rates. Our analysis suggested that the DENV2 Cosmopolitan genotype AO-clade likely originated from India (location posterior probability = 0.95) (Fig 3B). Both sequences from China can be found interspersed within Angolan genomes, revealing transmission of dengue virus from Angola to China. One strain (B09), a sample from mid-February 2019 collected in the southern province of Cunene clustered closely and with strong statistical support (posterior probability = 1.00) to P26, a genome sampled in Luanda province. Together with the high proportion of urban cases, this is suggestive of the establishment of the DENV2 Cosmopolitan genotype in the capital city of Luanda followed by spread to other provinces in Angola.

## Discussion

Our study reports molecular investigations of dengue suspected cases and genomic characterization of dengue confirmed cases reported to the Ministry of Health Angola. Genome sequencing of over a third of all DENV confirmed cases detected in Angola between January 2017 to July 2019, identified epidemic circulation of the DENV2 Cosmopolitan genotype. One DENV1 and 5 CHIKV infections were also detected during this period. Phylogenetic analysis of DENV revealed an introduction of this lineage in Angola around late October 2015 (February 2015 to June 2016), at least 1 year before its detection in the country.

The majority of the DENV detected cases occurred in urban settings, and the peak of confirmed cases between April and May 2018 occurred in months of high mosquito abundance in Luanda. However, confirmed cases were detected throughout the year, suggesting that Luanda’s climate provides year-round opportunities for the transmission of *Aedes aegypti*-borne viruses. The fact that most cases of DENV2 were detected in Luanda province but also throughout the coastal provinces of Angola is reminiscent of the patterns observed for the 2016 yellow fever outbreak [35] and probably reflects a combination of susceptible hosts, and suitable climatic conditions driving abundance of the *Aedes* spp. vector. However, while 13 of 18 provinces reported YFV cases in 2016, only 5 of 18 provinces had confirmed DENV2 cases between 2017–2019 (Fig 1B), suggesting either more limited circulation of DENV2 or more limited surveillance for DENV2 in comparison with YFV.

Human mobility plays a key role in the spread of *Aedes-*borne viruses and other emerging viruses [36,37]. Our phylogeographic analysis suggests that the DENV2 Cosmopolitan genotype was most likely introduced from India, a route of importation that is also supported by travel data [19]. It is important to stress that other unsampled locations could have acted as stepping-stone for arbovirus transmission. Based on the limited availability of genomes from the DENV2-II lineage (only 12 genomes are available from outside Angola in the clade that we consider, Fig 3B) these results should be interpreted with caution as it is possible that the virus seeding the recent epidemic derives from unsampled locations. Most air passengers flying to Angola initiate their travel in Brazil. However, despite frequent exchange of mosquito-borne viruses between the two countries (e.g. Zika and chikungunya virus), the DENV2-II has only recently been detected in Brazil [38], while the recently detected epidemic circulation of DENV2-III in Brazil [39] has not yet been observed in Angola. Moreover, two DENV2 sequences from China fall within the diversity of the Angolan sequences reported here. Similarly, infections in Chinese travelers visiting Angola have also been observed for YFV [40,41]. All cases shared high homology with a YFV strain from Angola, suggesting importation via returning travellers. Given the connectivity of Angola to South America and Asia, continued monitorization is needed to rapidly detect circulation of newly introduced dengue lineages and other viral pathogens, including SARS-CoV-2 for which Angola has been considered has highly vulnerable country [42].

Our phylogenetic analysis further suggests a single virus introduction into the country followed by an exponential population growth of the DENV2-II Angolan clade, with an estimated growth rate of around 2.59 new infections/individual/year. This represents a doubling epidemic time of 71 weeks (95% BCI, 33.8 weeks to 1.9 years), which is nearly 3-fold slower compared to estimates from DENV2-III sampled in Venezuela between 1997–2000 [43] and over 3-fold slower compared to estimates from DENV2 lineages sampled across the American continent [44]. Since the evolutionary rates of the different lineages are not significantly different, it is likely that slower population growth and more limited population dispersal outside of Luanda can both contribute to the more moderate spread of the virus within Angola in comparison with explosive outbreaks in the Americas. It is also possible that differences in Aedes spp. susceptibility and differences in population immunity in Angola may contribute to lower population growth rates compared to the Americas.

A quarter of Angola’s population reside in the capital city of Luanda, where the highest number of DENV acutely infected cases were detected. Many of the 7 million inhabitants in Luanda live in inadequate sanitation conditions which facilitate breeding of the *A*. *aegypti* mosquito vector. Thus, the increased detection of DENV in Luanda could indeed reflect higher incidence and more suitable conditions for arbovirus transmission in the city. Moreover, an entomological survey carried out between April 2014 to March 2015 revealed the presence of *A*. *aegypti* in all municipalities of the Luanda province [25]. Future public health prevention and control of dengue should rely on more effective vector control measures, particularly in Angola’s largest urban centers. Nevertheless, DENV detection in Luanda could reflect at least in part an ascertainment bias resulting from more challenging shipment and adequate storage of samples from provinces far away from Luanda [27]. Ongoing studies are attempting to analyse larger number of cases and evaluating strategies to de-centralize surveillance of viral outbreaks in provincial laboratories across Angola.

Reports of severe dengue in Africa are rare. We identified 9 cases exhibiting signs compatible with severe dengue disease in Angola. Sequential heterosubtypic dengue infections are associated with severe dengue [10]. Given previous circulation of DENV1 in 2013 in Luanda, the finding of severe dengue cases in Angola is not unexpected. During the previous 2013 DENV1 outbreak in Angola, one fatal case and one severe dengue case with clinically significant haemorrhagic manifestations were reported [20]. Moreover, undetected circulation of other serotypes in Angola cannot be ruled out. Indeed, DENV4 was detected in January 2014 in a traveller returning from Luanda [28]. We identified several aspects to improve serotype and genotype detection for arbovirus molecular surveillance in Angola. For example, 32% (*n* = 100) of samples from patients with suspected dengue were collected more than 6 days after symptom onset, at which point RT-qPCR testing is less effective because the patient is likely to no longer be acutely infected. Yet, some provinces may experience challenges in the shipment of samples to the reference laboratory because of inadequate road infrastructures. We are currently testing the use of chemically treated filter paper cards for transport of viable RNA to reduce the number of days between sample collection and testing and improve stability of RNA during transport.

The detection of a new DENV serotype is an important indicator of ongoing risk for future epidemics of severe dengue [7,10]. Intensified surveillance in Africa has resulted in several reports of severe dengue cases across the continent [11,12,20,45]. However, confirmation of dengue haemorrhagic fever typically requires laboratory tests such as haematocrits, platelet counts, virological and serological screening of patient samples with haemorrhagic symptoms. Laboratory equipment for these tests is not yet available in Angola. Timely diagnosis and implementation of dengue patient management strategies can help to provide more effective treatment of dengue disease. Between 2017 to 2020, INIS organized several short courses on arboviruses for healthcare professionals and public health staff across the country. Yet, increased awareness of dengue throughout the country and improved access to medical care should be further prioritized to increase reporting and help to lower fatality rates [46].

In conclusion, we present dengue epidemiological, clinical, and genetic findings from augmented routine surveillance efforts at the Ministry of Health in Angola. Expanding and de-centralizing the monitorization of arboviral epidemics across the country, increasing dengue awareness among healthcare personnel, identifying most common mosquito breeding sites in urban settings, implementing novel vector control interventions and dengue vaccination campaigns could help to reduce vector presence and DENV transmission in Angola.

## Materials and methods

### Ethics statement

Clinical samples (serum and blood) and associated demographic data were processed and analysed at the INIS, following guidelines of the recently established National Arbovirus Surveillance Laboratory in Angola from the Ministry of Health, Angola. Residual anonymized clinical diagnostic samples, with no or minimal risk to patients, were used for research and surveillance purposes as part of the National Arbovirus Surveillance in Angola within the terms of the National Ethics Committee, Ministry of Health, Angola.

### Context

The Republic of Angola, Angola, is located in southwestern Africa and has an area of 1,247 million km^2^ and an estimated 30,175,553 inhabitants in 2019 (https://www.ine.gov.ao). Sixty-two percent of its population live in urban areas. Angola is bordered by the Democratic Republic of Congo in the north, Zambia and Botswana in the East, and Namibia in the South. The country is organized into 18 provinces, 162 municipalities and 559 communes. The most populated province is Luanda province, with an estimated 7 million inhabitants (https://www.ine.gov.ao). The country’s climate is arid along the coast and in the south, with tropical and temperate climate in the interior of the country. Luanda’s climate is characterized by a rainy season from September to April, and a dry season from early May to the end of August, with average temperature of 24.4 C (minimum 20.4 °C in August and maximum of 26.9C in March) and average annual precipitation of 439 mm (concentrated between September to May).

### Dengue case definition and notification in Angola

Angolan Ministry of Health (MoH) National Arbovirus Surveillance Laboratory (NAS) guidelines for suspected DENV infection were introduced in April 2013 and include undifferentiated fever, maculopapular rash, myalgia, headache, arthralgia, retro-orbital pain, and vomiting. Severe cases were defined following the revised World Health Organization 2009 guidelines and include plasma leakage, bleeding and organ involvement [10]. Clinicians throughout the country were informed that the disease may be mild or occur in a more aggressive form which may be accompanied by epistaxis, gingival bleeding, hematemesis, haematuria, and melena. Serum samples from DENV suspected cases are submitted to INIS along with hand-written notification forms, which include information on sex, age, type of residence (urban or rural), clinical symptoms, date of onset of symptoms, travel history in the last two weeks, and other clinical laboratory tests conducted at local hospitals (thick and thin blood smears for malaria and hemograms). Suspected cases are not reported to INIS without accompanying samples for confirmation.

### Laboratory confirmation of DENV suspected cases

Based on the official MoH guidelines, 401 suspected DENV cases were reported to INIS between January 2017 and February 2019, of which only 351 cases had detailed epidemiological data. Only samples with epidemiological data were used in this study. Suspected cases were reported from 13 provinces (Fig 1). Serum samples from dengue suspected cases were screened for DENV RNA at INIS, Luanda. RNA was extracted from serum samples using QIAamp Viral RNA mini kits according to the manufacturer’s instructions. Dengue clinical symptoms often overlap with those from ZIKV and CHIKV [47]. Moreover, strong cross-reactivity in serological tests is often seen with other flaviviruses such as Zika, yellow fever, West Nile and Japanese encephalitis virus [48,49]. Thus, we conducted molecular diagnosis that accurately differentiates DENV, ZIKV and CHIKV infections using a Trioplex real-time RT-PCR assay [31]. For the DENV positive samples, we then used the CDC DENV1-4 RT-PCR assay [32] to identify specific DENV serotypes. Both RT-PCR assays were conducted on an Applied Biosystems 7500 Fast system. Sex, age, clinical data, province, municipality of sampling, type of residence and cycle threshold (CT) values for each RT-PCR confirmed case can be found in S1 Table.

### Portable on-site DENV nanopore genome sequencing

Sequencing was attempted on 25 RT-PCR positive samples using the Oxford Nanopore Technology MinION sequencer, according to protocols that have been described in detail previously [30,50]. 24 DENV2 complete or near complete viral genome sequences were generated. In brief, cDNA was reverse transcribed and subjected to a multiplex PCR designed to amplify overlapping fragments of ~1000bp that span the whole DENV2 coding region. A library was prepared with adaptor-ligated, barcoded PCR amplicons, and sequenced on the MinION. Bioinformatic generation of consensus sequences was conducted according to previously described protocols [30,50]. Following generation of consensus sequences, all sequences were aligned using Muscle v3.8 [51] and using DENV2 genome (Accession Number LC121816) as reference genome. All DENV2 sequences resulting from this study are available on GenBank Accession numbers: MW481670-MW481694.

### Compilation and curation of DENV2 genomic datasets

DENV2 genomes or partial genomes of at least 1,000 nucleotides in length were downloaded from GenBank in June 2019 [52] using an in-house python script (named as the *global dataset*). The search retrieved *n* = 1,584 sequences which were then classified into genotypes using an online phylogenetic-based tool [33] and confirmed with manual phylogenetic subtyping with RaXML v8 [53]. For manual phylogenetic subtyping, the global dataset and the new genome sequences from Angola were appended to a previously curated DENV2 reference dataset (n = 42) described in detail elsewhere [30]. Next, we prepared a separate dataset with all DENV2 sequences pertaining to the same DENV2 genotype as the one circulating in Angola. This resulted in a dataset of a total 38 complete genomes for subsequent analyses, including data from Angola (n = 25), China (n = 2), India (n = 10), and Ethiopia/Djibouti (n = 1). The resulting nucleotide dataset, named as DENV2 *Cosmopolitan dataset*, was then aligned using Muscle 3.8 [51]. Metadata on country/location of collection, date of sampling were manually retrieved from GenBank entries and directly from publications reporting the sequence data.

### Maximum likelihood phylogenetic analysis

Maximum likelihood (ML) phylogenetic trees were generated for the global and the Cosmopolitan datasets using RaXML v8 [53]. Statistical branch support was obtained using 1000 bootstrap replicates for the Cosmopolitan dataset. Analysis of temporal signal of this dataset was conducted using TempEst by regressing collection dates (format yyyy-mm-dd; a precision of 1 year or 1 month was used when exact dates were not available) and root-to-tip genetic distances [54]. One outlier sequence with an unusually low divergence for its sampling date (GenBank MH822954) was removed from subsequent analyses. Given the strong correlation between root-to-tip divergence and sampling dates for the Cosmopolitan dataset (Fig 2A), we conducted subsequent time-stamped Bayesian phylogenetic analyses using this dataset.

### Virus population dynamics

To estimate rate of population growth in the Angolan lineages and the lineages paraphyletic to the Angolan clade (AO) from the Indian subcontinent and from Ethiopia/Djibouti, hereafter named as pre-AO lineages, we used a recently described nested Bayesian coalescent approach [34] that fits a constant-logistic demographic model [55] to the genealogy excluding the AO lineage. The initial constant phase accommodates the longer branches from pre-AO lineages as shown in Fig 2B. Nested within this model, we specified a separate logistic growth model for the AO lineage. We compare the fit of the nested constant-logistic to four other coalescent models: constant population size, exponential population size, logistic population size and the Bayesian skyline. Model selection results were conducted with path-sampling and sampling stone approaches [56] in BEAST v1.10 [57].

### Bayesian phylogeographic analysis

Bayesian molecular clock analyses were conducted in BEAST v1.10 with both strict and uncorrelated relaxed molecular clock models [57,58]. We used a strict molecular clock that assumes a single evolutionary rate across all phylogenetic branches of the DENV2 Cosmopolitan genotype, after a preliminary relaxed clock model analysis revealed a coefficient of variation close to zero (median 0.17, 95% BCI: 7.6 x 10^−5^ to 0.34). To estimate ancestral locations throughout DENV2 Cosmopolitan genotype history, we used an asymmetric discrete trait phylogenetic model that takes into account shared ancestry of virus genomes and phylogenetic uncertainty [59]. Strict molecular clock phylogeographic analyses with the best fitting coalescent model were run in duplicate in BEAST v1.10 for 50 million MCMC steps [57]. Convergence of the MCMC chains was inspected using Tracer version 1.7.1 [60], and after removing 10% of the posterior trees as burn-in, a maximum clade credibility tree were generated with TreeAnnotator v.1.7.1 [57] and visualized in FigTree v.1.4.3 [61].

## Supporting information

S1 Table Metadata associated with DENV RT-qPCR positive cases and sequenced samples analysed at INIS between January 2017 and February 2019. (XLSX)

S2 Table Epidemiological description of dengue cases reported from January 2017 to February. 2019 in Angola. (DOCX)

S3 Table Results for the coalescent tree prior model selection analysis. (XLSX)
