# Supplementary material for: Molecular and genomic investigation of an urban outbreak of dengue virus serotype 2 in Angola, 2017–2019
Source: PLoS Negl Trop Dis. 2022 May 18;16(5):e0010255. doi: 10.1371/journal.pntd.0010255 (PMC9166355; doi:10.1371/journal.pntd.0010255)
Supplement: S2 Table — (DOCX) [file pntd.0010255.s002.docx]

**Table S1**. Epidemiological description of dengue cases reported from January 2017 to February. 2019 in Angola.

| **Variables** | **Total** | **Negative** | **Positive** | **OR** | **95 % CI** | **Significance P** |
| --- | --- | --- | --- | --- | --- | --- |
| Female | 154 | 125 | 29 | 0.87 | 0.51–1.47 | 0.595 |
| Male | 194 | 153 | 41 |  |  |  |
| <18 | 206 | 170 | 36 | 0.67 | 0.39–1.14 | 0.1404 |
| >18 | 142 | 108 | 34 |  |  |  |
| Urban | 185 | 126 | 59 | 7.9 | 2.77–22.8 | 0.0001 |
| Rural | 72 | 68 | 4 |  |  |  |
| < 6 days | 228 | 174 | 54 | 2.13 | 1.1– 4.03 | 0.0204 |
| > 6 days | 110 | 96 | 14 |  |  |  |
